# Genetic epidemiology of blood type, disease and trait variants, and genome-wide genetic diversity in over 11,000 domestic cats

**DOI:** 10.1371/journal.pgen.1009804

**Published:** 2022-06-16

**Authors:** Heidi Anderson, Stephen Davison, Katherine M. Lytle, Leena Honkanen, Jamie Freyer, Julia Mathlin, Kaisa Kyöstilä, Laura Inman, Annette Louviere, Rebecca Chodroff Foran, Oliver P. Forman, Hannes Lohi, Jonas Donner

**Affiliations:** 1 Wisdom Panel Research Team, Wisdom Panel, Kinship, Portland, Oregon, United States of America; 2 Department of Medical and Clinical Genetics, University of Helsinki, Helsinki, Finland; 3 Department of Veterinary Biosciences, University of Helsinki, Helsinki, Finland; 4 Folkhälsan Research Center, Helsinki, Finland; University of Cambridge, UNITED KINGDOM

## Abstract

In the largest DNA-based study of domestic cats to date, 11,036 individuals (10,419 pedigreed cats and 617 non-pedigreed cats) were genotyped via commercial panel testing elucidating the distribution and frequency of known disease, blood type, and physical trait associated genetic variants across cat breeds. This study provides allele frequencies for many disease-associated variants for the first time and provides updates on previously reported information with evidence suggesting that DNA testing has been effectively used to reduce disease associated variants within certain pedigreed cat populations over time. We identified 13 disease-associated variants in 47 breeds or breed types in which the variant had not previously been documented, highlighting the relevance of comprehensive genetic screening across breeds. Three disease-associated variants were discovered in non-pedigreed cats only. To investigate the causality of nine disease-associated variants in cats of different breed backgrounds our veterinarians conducted owner interviews, reviewed clinical records, and invited cats to have follow-up clinical examinations. Additionally, genetic variants determining blood types A, B and AB, which are relevant clinically and in cat breeding, were genotyped. Appearance-associated genetic variation in all cats is also discussed. Lastly, genome-wide SNP heterozygosity levels were calculated to obtain a comparable measure of the genetic diversity in different cat breeds. This study represents the first comprehensive exploration of informative Mendelian variants in felines by screening over 10,000 pedigreed cats. The results qualitatively contribute to the understanding of feline variant heritage and genetic diversity and demonstrate the clinical utility and importance of such information in supporting breeding programs and the research community. The work also highlights the crucial commitment of pedigreed cat breeders and registries in supporting the establishment of large genomic databases, that when combined with phenotype information can advance scientific understanding and provide insights that can be applied to improve the health and welfare of cats.

## Author summary

Domestic cats are one of the world’s most popular companion animals, of which pedigreed cats represent small unique subpopulations. Genetic research on pedigreed cats has facilitated discoveries of heritable conditions resulting in the availability of DNA testing for studying and managing inherited disorders and traits in specific cat breeds. We have explored an extensive study cohort of 11,036 domestic cat samples representing pedigreed cats of 90 breeds and breed types. This work provided insight into the heritage of feline disease and trait alleles. We gained knowledge on the most common and relevant genetic markers for inherited disorders and physical traits, and the genetic determinants of the clinically relevant AB blood group system. We also used a measure of genetic diversity to compare inbreeding levels within and between breeds. This information can help support sustainable breeding goals within the cat fancy. Direct-to-consumer genetic tests help to raise awareness of various inherited single gene conditions in cats and provide information that owners can share with their veterinarians. In due course, ventures of this type will enable the genetics of common complex feline disease to be deciphered, paving the way for precision healthcare with the potential to ultimately improve welfare for all cats.

## Introduction

Domestic cats are valued companions to humans, with owners seeking high quality veterinary care to support long-term wellness and positive outcomes for disease treatment. Cat genomes are structured in a similar way to humans, with 90% of their genes having a human homologue, and cats and humans also suffer from many similar diseases [1]. Domestic cats, like dogs, can serve as naturally occurring models for many human diseases, in which the development of therapeutic treatments may be helpful for both veterinary and human patients [1–6]. Genomic medicine, encompassing the understanding of individual genetic variability in disease risk and the customization of healthcare by patient subgroups, is now feasible and likely to become a future standard-of-care in veterinary medicine and the care of companion animals, including cats [1]. Fueling this development for felines specifically are improved genomic resources and technologies, combined with a steady increase in the discovery of Mendelian disease and trait variants [7,8]. Genetic testing is a form of genomic medicine when used for diagnosing diseases or traits of clinical relevance, as the results are patient-specific and can potentially be used to tailor treatment to the disease and the patient [9]. Direct-to-consumer genetic testing is now readily available, and further empowers owners to proactively invest in potentially changing the medical care for their pet.

Domestication of cats occurred approximately 10,000 years ago [10], with pedigreed cats emerging over the last 150 years representing genetically unique domestic cat subpopulations. The majority of the approximately 100 cat breeds, breed types (or varieties of the breed) known today, including several new breeds being developed, represent breeds that are less than 75 years old. The genetic research of isolated populations in humans, dogs, and more recently cats has vastly improved identification of genetic disease and trait variants for use in genetic diagnostics and medicine. As an example, through studies of pedigreed cats, researchers have identified disease-associated variants for both diseases that are affecting cats worldwide such as Polycystic Kidney Disease and rare disorders within a specific breed such as Hypotrichosis in Birman cats [11–19]. Moreover, studies of cat breeds have enabled discovery of the genetic variants determining blood types of the AB blood group system that associate with neonatal isoerythrolysis and hemolytic transfusion reactions; and many of the physical traits, which are an integral part of breed development [8,20–25]. Research on genetically isolated populations has further highlighted that reduced genetic diversity, because of isolation and generations of selective breeding to consistently produce animals with uniform appearance, can manifest as an increase in the number of health conditions that are identified [26–29]. For more than a decade, it has been common practice to eradicate disease-associated variants from pedigreed cat breeding populations using DNA testing. However, the focus on eradicating single DNA variants from a breed could contribute to severe loss of genetic diversity, especially if implemented strictly instead of thoughtfully [30]. Improved genomic perspective on domestic cats, and the breeds’ level of genetic diversity, have propelled a growing number of breeders to incorporate breeding strategies for sustaining genetic diversity and increasing it through crossbreeding. Crossbreeding in cat breeding is also used to introduce a trait that is new to the breed. In addition, the cat breeds that are becoming increasingly more common have a hybrid origin such as the world’s most popular cat breed, the Bengal, that is a result of breeding an Asian Leopard Cat with the domestic cat.

Our previous work has elucidated that the comprehensive screening of genetic variants in dogs is convenient and justified as it provides information to support breeding programs, veterinary care and health research [31]. Further large-scale multiplex screening approaches were taken to characterize canine disease heritage and the relative frequency and distribution of disease-associated variants across breeds and to explore the frequency of known canine appearance-associated variants among dog breeds [32,33]. As we will demonstrate, such efforts are now equally feasible in cats and hold comparable promise for gaining insight into the genetic epidemiology of feline diseases and traits to better inform feline breeding decisions and establish the foundation for precision medicine of individuals, populations, and breeds.

This study represents the first comprehensive genetic evaluation of known feline disease and trait variants through the examination of 87 variants in 10,419 pedigreed cats and 617 non-pedigreed cats. These results provide a first glance into feline variant heritage across cat breeds and underscore the importance of large-scale population screening studies in improving veterinary diagnostics, breeding programs, and health recommendations for all cats.

## Materials and methods

### Ethics statement

Feline DNA was obtained by Wisdom Panel as owner submitted, non-invasive cheek swab samples or was collected by certified veterinary clinics as cheek swab and blood samples in accordance with international standards for animal care and research. All cat owners provided consent for the use of their cat’s DNA sample in scientific research. University biobank samples were collected under the permit ESAVI/6054/04.10.03/2012 by the Animal Ethics Committee of the State Provincial Office of Southern Finland, Hämeenlinna, Finland.

### Study sample population

The cat study population consisted of 10,419 pedigreed cats and 617 non-pedigreed cats whose samples were obtained during the development and provision of the commercially available MyCatDNA and Optimal Selection Feline tests (Wisdom Panel, Helsinki, Finland and Wisdom Panel, Vancouver, WA, USA, respectively) between 2016 and 2021. The 10,419 pedigreed cat samples represented 90 breeds and breed types (or variants of the breed) with 60 (66.6%) breeds and breed types represented by 15 or more individuals (S1 Table).

The breed of a cat was reported by its owner with accompanying registration information confirming the cat was registered with The International Cat Association (TICA), Fédération Internationale Féline (FiFe), The Cat Fanciers’ Association (CFA), or World Cat Federation (WCF) standards. Additional breeds not yet recognized by any major breed registry but with an established community of breed hobbyists were also considered breeds for the purposes of this study. The non-pedigreed cat sample set consisted of mixed breeds, breed crosses, or random-bred cats. The tested cats were most often from the United States of America (54.9%), while cats from Finland (17.4%), Canada (5.3%), United Kingdom (3.5%), Norway (3.5%) and Sweden (3.3%), Russia (2.5%) and France (1%) represented other notable subgroups (>1% of the sample).

### Microarray development and validation

A custom genotyping microarray for selected feline disease and trait associated variants (S2 Table) was developed based on the Illumina Infinium XT platform (Illumina, Inc., San Diego, CA, USA), commercially available as the Wisdom Panel Complete for Cats / MyCatDNA / Optimal Selection Feline tests. The microarray was designed and validated for use following the same protocol and principles as previously described for canines [21]. Firstly, public databases [8] and searches of the scientific literature were used to identify likely causal variants for feline Mendelian disorders and traits. Secondly, genotyping assays for the identified variants were designed according to the manufacturer’s guidelines (Illumina, Inc.). At least three technical replicates of each target sequence were included in the array design. Thirdly, the validation of each specific disease and trait assay was completed with the use of control samples of known genotype or synthetic oligonucleotides in the case of rare conditions for which no control samples were available. In addition, owner-provided photographs contributed to phenotypic validation of trait variant tests.

### Genotyping

Microarray genotyping analyses were carried out following manufacturer-recommended standard protocols for the Illumina XT platform (Illumina, Inc.). All genotype data from samples with call rates below 98% of the analyzed markers were discarded to ensure high quality data and all disease-associated variant calls were confirmed by manual review. Only disease and trait variants reaching 100% sensitivity and specificity upon testing with natural control samples that are homozygous/heterozygous for the disease or trait associated variant (70/87 variants), or validated using synthetic control oligonucleotides (17/87 variants), were considered for reporting. Genome-wide genetic diversity was measured as a percentage of heterozygous SNPs across a set of 7,815 informative SNPs. Data available at Dryad Digital Repository [34]. Selected disease-associated variant findings were genotyped using standard capillary sequencing on an ABI3730xl DNA Analyzer platform (Thermo Fisher Scientific, Waltham, MA, USA) as a secondary technology to provide further validation of results that were unexpected for the breed. Sequencing was performed at the Sanger Sequencing Unit of the Finnish Institute of Molecular Medicine (FIMM). The DNA extractions and PCR-product preparation and purification were carried out as previously described in detail [31] using ~20 ng of genomic template DNA and an Amplitaq Gold Master Mix-based protocol according to the manufacturer’s instructions (Applied Biosystems, Waltham, MA, USA).

### Clinical validations

Medical history information for genetically affected cats was collected through interviews with cat owners and veterinary clinicians. Clinical examinations were performed for confirmation of a Factor XII deficiency diagnosis by collecting a blood sample for an activated partial thromboplastin time screening test through IDEXX Laboratories (IDEXX Europe B.V., Hoofddorp, The Netherlands). Progressive Retinal Atrophy diagnosis was confirmed via ophthalmic examination performed by an ECVO (European College of Veterinary Ophthalmologists) board-certified veterinary specialist. A set of blood type determination data was available through the records of the former Genlab Niini (Helsinki, Finland). All additional phenotype information (clinical or trait) and documentation was obtained through voluntary owner submissions.

## Results

### Overview of genotyping results

A total of 11,036 domestic cats, mainly pedigreed cats (N = 10,419) representing 90 breeds and breed types (or variants of the breed) and an additional 617 non-pedigreed cats, were successfully genotyped on the custom array (the sample failure rate for DNA extracted from buccal swabs was <1%). Genetic screening included genotyping of 7,815 informative SNP markers across the genome and 87 variants associated with blood type, diseases, and/or physical appearance; 83 of which were evaluated in the entire 11,036 cat study sample and four more recently included variants to the genotyping platform that were screened for in a subset of 2,186 samples (Tables 1 and S1–S4). We observed 57 (65.5%) of the 87 tested variants at least once in this study cohort, including 22 (38.6%) disease-associated variants and 35 (61.4%) variants associated with an appearance-associated trait or blood type (Tables 1 and S2–S4). The maximum number of disease-associated variants observed in any one individual was four. We observed that 2,480 (22.5%) of the tested cats had at least one disease-associated variant present and 452 (4.1%) of the tested cats were potentially at risk for at least one health condition, in accordance with the disorders’ modes of inheritance. The maximum number of different disease-associated variants present in a single breed was 9; this was observed in the Maine Coon, which was the breed that was represented by the most individuals (N = 1971) tested in this dataset. Three genetic variants for the diseases Hyperoxaluria Type II [35], Lipoprotein Lipase Deficiency [36] and Myotonia Congenita [37], were solely observed in non-pedigreed cat samples.

While we observed several disease-associated variants in the breeds with documented occurrence, we also detected 13 disease-associated variants in additional breeds or breed types in which the variants had not previously been reported. For each additional breed finding, extensive review of the breed information was performed, including use of the proprietary Wisdom Panel cat breed determination algorithm where necessary. Details of the variants found in additional breeds are listed in Table 2. Within these additional breeds, individuals that were genetically affected (having one copy of a dominant variant or two copies of a recessive variant) were identified and flagged for clinical follow-up. Individuals that were genetically affected for candidate disease-associated variants previously presented by a case study, were also shortlisted for follow-up.

**Table 1:** Prevalence and frequency of the tested disease, blood type and trait -associated variants in all cats.

Disease	OMIA ID	Gene	OMIA variant ID	MOI	Derived Allele Frequency (%) in all cats	Cats genotyped with 0 copies	Cats genotyped with 1 copy	Cats genotyped with 2 copies
Acute Intermittent Porphyria—5 variants	001493–9685	*HMBS*	596, 530, 135, 402, 501	AD	*not present*	11036		
Autoimmune Lymphoproliferative Syndrome	002064–9685	*FASLG*	613	AR	*not present*	11036		
Burmese Head Defect	001551–9685	*ALX1*	550	AR	0.02%	11034	2	
Congenital Adrenal Hyperplasia	001661–9685	*CYP11B1*	117	AR	*not present*	10990		
Congenital Erythropoietic Porphyria	001175–9685	*UROS*	137	AR	*not present*	11036		
Congenital Myasthenic Syndrome	001621–9685	*COLQ*	944	AR	0.1%	11025	11	
Cystinuria Type 1A	000256–9685	*SCL3A1*	141	AR	*not present*	11036		
Cystinuria Type B	002023–9685	*SLC7A9*	143	AR	0.1%	11025	10	
Cystinuria Type B—2 variants	002023–9685	*SCL7A9*	142, 144	AR	*not present*	11036		
Dihydropyrimidinase Deficiency	001776–9685	*DPYS*	125	AR	*not present*	11036		
Factor XII Deficiency	000364–9685	*F12*	533	AR	1.3%	10640	264	12
Factor XII Deficiency*	000364–9685	*F12*	147	AR	7.0%	1894	261	22
Familial Episodic Hypokalemic Polymyopathy	001759–9685	*WNK4*	312	AR	0.02%	11031	5	
Glutaric Aciduria Type II	001457–9685	*ETFDH*	1439	AR	*not present*	11036		
Glycogen Storage Disease	000420–9685	*GBE1*	742	AR	0.01%	11035	1	
GM1 Gangliosidosis	000402–9685	*GLB1*	126	AR	*not present*	11036		
GM2 Gangliosidosis	001427–9685	*GM2A*	496	AR	*not present*	11034		
GM2 Gangliosidosis Type II (Discovered in the Burmese)	001462–9685	*HEXB*	381	AR	0.01%	11034	2	
GM2 Gangliosidosis Type II—2 variants (Discovered in domestic cats)	001462–9685	*HEXB*	741, 309	AR	*not present*	11036		
Hemophilia B -2 variants	000438–9685	*F9*	127, 310	X-linked	*not present*	11036		
Hyperoxaluria Type II	000821–9685	*GRHPR*	383	AR	0.04%	11028	8	
Hypertrophic Cardiomyopathy; HCM (Discovered in the Ragdoll)	000515–9685	*MYBPC3*	902	AD	0.2%	10991	42	1
Hypertrophic Cardiomyopathy; HCM (Discovered in the Maine Coon)	000515–9685	*MYBPC3*	901	AD	0.9%	10858	169	9
Hypotrichosis	001949–9685	*FOXN1*	1319	AR	*not present*	11018		
Lipoprotein Lipase Deficiency	001210–9685	*LPL*	131	AR	0.02%	11032	4	
MDR1 Medication Sensitivity	001402–9685	*ABCB1*	322	AR	0.6%	10910	123	3
Mucopolysaccharidosis Type I	000664–9685	*IDUA*	500	AR	*not present*	10952		
Mucopolysaccharidosis Type VI	000666–9685	*ARSB*	132	AR	*not present*	11036		
Mucopolysaccharidosis Type VII—2 variants	000667–9685	*GUSB*	133, 139	AR	*not present*	11036		
Myotonia Congenita	000698–9685	*CLCN1*	408	AR	0.00%	11035		1
Osteochondrodysplasia and Earfold	000319–9685	*TRPV4*	140	AD	0.4%	10945	90	1
Polycystic Kidney Disease; PKD	000807–9685	*PKD1*	314	AD	0.1%	10965	11	
Progressive Retinal Atrophy (Discovered in the Bengal)	002267–9685	*KIF3B*	1191	AR	1.1%	11765	241	2
Progressive Retinal Atrophy (Discovered in the Persian)*	001222–9685	*AIPL1*	1214	AR	*not present*	2178		
Progressive Retinal Atrophy; rdAc-PRA	001244–9685	*CEP290*	384	AR	1.1%	10792	227	17
Pyruvate Kinase Deficiency; PK-def	000844–9685	*PKLR*	899	AR	2.9%	10286	588	18
Sphingomyelinosis; Niemann-Pick C1	000725–9685	*NPC1*	134	AR	*not present*	11036		
Sphingomyelinosis; Niemann-Pick C2	002065–9685	*NPC2*	420	AR	*not present*	10969		
Spinal Muscular Atrophy (Discovered in the Maine Coon)	002389–9685	*LIX1*	649	AR	0.1%	11016	13	
Vitamin D-Dependent Rickets	001661–9685	*CYP27B1*	315	AR	*not present*	11025		
**Blood type, Allele Designation**								
Blood type B, b^1^ (2019 Typing panel)	000119–9685	*CMAH*	119	AR	12.9%	8626	1981	428
Blood type B, c (2019 Typing panel)	000119–9685	*CMAH*	799	AR	1.5%	10707	318	11
Blood type B, b^2^ (2019 Typing panel)	000119–9685	*CMAH*	800	AR	1.6%	10707	301	28
Blood type B, b^3^ (2019 Typing panel)*	000119–9685	*CMAH*	1062	AR	2.4%	2081	90	8
**Trait, Allele Designation**								
Albinism, c^a^ (Discovered in Oriental breeds)	000202–9685	*TYR*	494	AR	*not present*	11022		
Amber, e (Discovered in the Norwegian Forrest Cat)	001199–9685	*MC1R*	123	AR	0.03%	10982	4	1
Chocolate, b	001249–9685	*TYRP1*	379	AR	13.2%	8762	1636	638
Cinnamon, b^l^	001249–9685	*TYRP1*	306	AR	4.0%	10308	4264	2402
Charcoal, A^Pb^ (Discovered in the Bengal) -2 variants	000201–9685	*ASIP*	1451, 1450	intermediate	1.9%	10660	328	48
Colorpoint, c^b^ (Discovered in the Burmese)	000202–9685	*TYR*	121	AR	6.8%	9860	859	317
Colorpoint, c^s^ (Discovered in the Siamese)	000202–9685	*TYR*	122	AR	30.5%	6537	2247	2238
Dilution, d	000031–9685	*MLPH*	495	AR	41.6%	4364	4370	2402
Gloving, w^g^	001580–9685	*KIT*	620	AR	12.7%	8661	1943	432
Hairlessness, re^hr^ (Discovered in the Sphynx)	001583–9685	*KRT71*	382	AR	3.9%	10774	232	30
Long Hair, M1 (Discovered in the Ragdoll)	000439–9685	*FGF5*	595	AR	3.10%	10394	576	64
Long Hair, M2 (Discovered in the Norwegian Forrest Cat)	000439–9685	*FGF5*	311	AR	2.7%	10540	390	103
Long Hair, M3 (Discovered in the Ragdoll and Maine Coon)	000439–9685	*FGF5*	498	AR	12.0%	8253	1661	414
Long Hair, M4 (Discovered in many breeds)	000439–9685	*FGF5*	130	AR	38.10%	5188	3273	576
Partial and Full White, w^s^, W	001737–9685	*KIT*	732	AD	20.8%	7478	2504	1046
Polydactyly, Hw	000810–9685	*LMBR1*	432	AD (incomplete)	1.30%	10774	232	30
Polydactyly, UK1	000810–9685	*LMBR1*	433	AD (incomplete)	0.02%	11033	2	1
Polydactyly, UK2	000810–9685	*LMBR1*	434	AD (incomplete)	*not present*	11036		
Rexing, r (Discovered in the Cornish Rex)	001684–9685	*LPAR6*	522	AR	1.0%	10913	13	108
Rexing, re^dr^ (Discovered in the Devon Rex)*	001581–9685	*KRT71*	380	AR	5.5%	1986	33	100
Russet, e^r^ (Discovered in the Burmese)	001199–9685	*MC1R*	561	AR	*not present*	10953		
Short Kinked Tail, jb (Discovered in the Japanese Bobtail)	001987–9685	*HES7*	145	AD (incomplete)	0.3%	10993	17	26
Short Tail, C1199del (Discovered in the Manx)	000975–9685	*T*	525	AD	0.3%	10776	65	
Short Tail, T988del (Discovered in the Manx)	000975–9685	*T*	523	AD	1.8%	10900	99	
Short Tail, C1169del (Discovered in the Manx)	000975–9685	*T*	524	AD	0.06%	10883	14	
Solid Color, a	000201–9685	*ASIP*	493	AR	56.2%	3187	3280	4556
**Other genotyped Variants** ^#^								
Congenital Erythropoietic Porphyria	001175–9685	*UROS*	137	-	0.7%	10880	148	8
Mucopolysaccharidosis Type VI Modifier	000666–9685	*ARSB*	1320	-	2.5%	10537	443	56
Blood type B related	000119–9685	*CMAH*	430	-	12.9%	7937	2589	509
Blood type B related	000119–9685	*CMAH*	1431	-	6.5%	9844	953	238
Blood type B related	000119–9685	*CMAH*	118	-	16.3%	8621	1985	430
Blood type B related	000119–9685	*CMAH*	801	-	25.2%	6554	3398	1081
Blood type B related	000119–9685	*CMAH*	1446	-	30.2%	5901	3613	1522
Blood type B related	000119–9685	*CMAH*	120	-	12.8%	8614	2021	399

* The allele frequencies and genotypes are based on a subset of 2,186 samples (19.8% of the full study sample) screened for this variant.

# Genetic screening for these variants is available, but the predictive value is low. Abbreviations: MOI; mode of inheritance, AR; autosomal recessive, AD; autosomal dominant.

**Table 2:** Summary of disease-associated variant findings in additional breeds and breed types.

Disease	OMIA ID	Gene; OMIA variant ID	Derived allele prevalence in previously known breeds (with % for breeds with >15 genotyped cats)	Cats in additional breed(s) with Derived allele prevalence in additional breed(s) (with % for breeds with >15 individuals genotyped)
Cystinuria Type B	002023–9685	*SLC7A9*; 143	**non-pedigree cat (0/617) 0.0%** **Maine Coon (3/1971) 0.2%** **Sphynx (0/547) 0.0%**	**Maine Coon Polydactyl (3/150) 0.02%** **Siberian (4/559) 0.7%**
Factor XII Deficiency*	000364–9685	*F12*; 147	**non-pedigreed cat (21/90) 23.3%** **Bengal (63/311) 20.3%** **Maine Coon (53/497) 10.6%** **Siamese (2/21) 9.5%**	Balinese (2/9) Bombay (1/1) **British Shorthair (4/96) 4.1%** Cashmere (2/3) **Devon Rex (17/97) 17.5%** Donskoy (2/4) Elf (1/8) Exotic Shorthair (4/9) **Highlander (1/94) 1.0%** Himalayan (1/2) **Lykoi (3/19) 15.8%** **Maine Coon Polydactyl (1/43) 2.3%** Minuet (1/4) Minuet Longhair (3/11) **Munchkin (1/15) 6.7%** Munchkin Longhair (2/5) Neva Masquerade (1/12) Oriental Longhair (1/2) Oriental Shorthair (2/12) Persian (1/5) Peterbald (3/6) **Ragdoll (36/298) 12.0%** Savannah (5/12) **Scottish Fold Shorthair (3/31) 9.7%** **Scottish Straight (1/21) 4.8%** Scottish Straight Longhair (1/5) **Selkirk Rex (8/17) 47.0%** **Selkirk Rex Longhair (6/16) 37.5%** **Siberian (5/97) 5.1%** **Sphynx (9/110) 8.1%** Tennessee Rex (10/13) Turkish Angora (5/13)
Factor XII Deficiency	000364–9685	*F12*; 533	**non-pedigree cat (29/606) 4.8%** **Bengal (1/1668) 0.06%** **Maine Coon (195/1964) 9.9%** **Siamese (2/144) 1.4%**	**American Shorthair (1/48) 2.0%** **Balinese (1/76) 1.3%** **Cymric (2/16) 12.5%** **Highlander (1/201) 0.5%** **Himalayan (1/16) 6.3%** **Maine Coon Polydactyl 3.02% (9/149) 6.0%** **Manx (2/29) 6.9%** Minuet (1/11) **Munchkin (2/38) 5.2%** Munchkin Longhair (1/10) **Ragdoll (1/1110) 0.01%** **Savannah (5/78) 6.4%** **Tennessee Rex (22/32) 68.7%**
GM2 Gangliosidosis Type II	001462–9685	*HEXB*; 381	**Burmese (1/113) 0.9%**	**non-pedigreed cat (Burmese mix) (1/617) 0.7%**
Hypertrophic Cardiomyopathy; HCM (Discovered in the Maine Coon)	000515–9685	*MYBPC3*; 901	**Maine Coon (163/1971) 0.8%**	**Maine Coon Polydactyl (10/150) 6.7%** **non-pedigree cat (2/617) 0.3%** Pixiebob Longhair (1/12) **Siberian (1/559) 0.2%**
Hypertrophic Cardiomyopathy; HCM (Discovered in the Ragdoll)	000515–9685	*MYBPC3*; 902	**Ragdoll (33/1115) 3.0%**	**American Bobtail Longhair (4/35) 11.4%** **American Bobtail Shorthair (1/9)** **Highlander (1/201) 0.5%** **Munchkin 1.25% (1/40) 2.5%** **RagaMuffin (2/118) 1.7%** **non-pedigree cat (1/617) 0.2%**
MDR1 Medication Sensitivity	001402–9685	*ABCB1*; 322	**non-pedigree cat (5/617) 0.8%** **Ragdoll (0/1115) 0.0%** **Russian Blue (0/64) 0.0%** **Siamese (1/146) 0.7%**	**Balinese (2/76) 2.6%** **Maine Coon (107/1971) 5.4%** **Maine Coon Polydactyl (10/150) 6.7%** **Turkish Angora (1/110) 0.9%**
Osteochondrodysplasia and Earfold	000319–9685	*TRPV4*; 140	Scottish Fold Longhair (13/14) **Scottish Fold Shorthair (72/76) 94.7%**	**non-pedigree cat (Scottish Fold mix) (6/617) 1.0%**
Polycystic Kidney Disease; PKD	000807–9685	*PKD1*; 314	**Persian (0/118) 0.0%** **Exotic Shorthair (1/68) 1.5%** **Scottish Fold Shorthair (2/76) 2.6%** **Siberian (1/559) 0.2%** **Ragdoll (1/1114) 0.09%**	**Maine Coon (5/1966) 0.3%** **Scottish Straight (1/61) 1.6%**
Progressive Retinal Atrophy (Discovered in the Bengal)	002267–9685	*KIF3B*; 1191	**Bengal (322/1703) 18.9%**	**Highlander (7/201) 3.5%** **Highlander Shorthair (2/30) 6.7%** **Savannah (1/80) 1.3%** **non-pedigree cat (Bengal Mix) (1/607) 0.2%**
Progressive Retinal Atrophy; rdAc-PRA (Discovered in the Abyssinian)	001244–9685	*CEP290*; 384	**Abyssinian (18/167) 10.7%** **Balinese (15/76) 19.7%** **Cornish Rex (31/106) 29.2%** **Oriental Shorthair (41/178) 23.0%** **Pederbald (8/17) 47.0%** **Siamese (44/146) 30.1%** **Somali (6/47) 12.7%**	**American Shorthair (1/49) 2.0%** **Devon Rex (14/447) 3.1%** **Donskoy (1/17) 5.9%** **European Shorthair (1/91) 1.1%** **Havana Brown (1/30) 3.3%** **Highlander (1/201) 0.5%** **Maine Coon (2/1971) 0.1%** **Manx (3/30) 10.0%** **non-pedigree cat (14/617) 0.2%** **Oriental Longhair (17/51) 33.3%** Pixiebob Longhair (1/12) **Ragdoll (11/1115) 1.0%** **Savannah (10/80) 12.5%** **Scottish Fold Shorthair (2/76) 2.6%** **Sphynx (1/547) 0.2%** **Tennessee Rex (1/35) 2.9**%
Pyruvate Kinase Deficiency; PK-def	000844–9685	*PKLR*; 899	**Abyssinian (9/163) 5.5%** **Somali (11/78) 14.1%** **Bengal (190/1692) 11.2%** **Egyptian Mau (12/51) 23.5%** **Laperm (1/35) 2.9%** **non-pedigree cat (15/607) 2.9%** **Norwegian Forest Cat (4/121) 3.3%** **Maine Coon (280/1955) 14.3%** **Savannah (11/78) 14.1%** **Singapura (2/45) 4.4%**	Caracat (1/1) Chausie (1/6) **European Shorthair (1/91) 1.1%** **Highlander (4/201) 2.0%** **Highlander Shorthair (2/30) 6.7%** **Lykoi (7/104) 6.7%** **Maine Coon Polydactyl (30/150) 20.2%** **Minuet Longhair (2/24) 8.3%** **Munchkin (2/39) 5.1%** **Neva Masquerade (1/23) 4.3%** **Pixiebob (5/19) 26.3%** Pixiebob Longhair (4/11) Toyger (1/13)
Spinal Muscular Atrophy	002389–9685	*LIX1*; 649	**Maine Coon (11/1971) 0.6%**	**Highlander (1/201) 0.5%** **Maine Coon Polydactyl (1/150) 0.7%**

*There was a subset of 2,186 samples (19.8% of the full study sample) screened for this variant.

### Genetic epidemiology of the common AB blood group system across breeds and breed types

The major feline AB blood groups including blood type A, blood type B and the rare blood type AB are caused by functional differences in the cytidine monophospho-N-acetylneuraminic acid hydroxylase enzyme, encoded by the *CMAH* gene, that impact the ability of the enzyme to convert sialic acid N-acetylneuraminic acid (Neu5Ac) to N-glycolylneuraminic acid (Neu5Gc) on erythrocytes [20–25]. For purpose-bred cats, the current convention (2019 typing panel recommendation) [23,24], proposes that genetic testing for blood types A, B and AB should be based on panel testing of the four following variants c.179G>T, c.268T>A, c.364C>T and c.1322delT of *CMAH*. In this study, we obtained genotypes for ten blood type-associated *CMAH* variants; c.1-53delGTCGAAGCCAACGAGCAA (18bp indel), c.139C>T, c.142G>A, c.179G>T, c.187A>G, c.268T>A, c.327A>C, c.364C>T, c. 1322delT and c.1603G>A. All 11,036 cats were genotyped for nine of these *CMAH* variants, while c.1322delT was more recently added to the genotyping platform providing genotype results for a subset of 2,186 cats (19.7%). To evaluate the suitability of the proposed ‘2019 typing panel’ as the blood type genotyping scheme, we compared the obtained genotype information to the immunological blood type results available for 220 cats of this study cohort (S5 Table). We found that blood types assigned according to the cat’s 2019 typing panel-based genotype showed a 99.1% concordance with immunologically determined blood types. One Ragdoll with the c/c genotype indicative of blood type AB [20,23,24], had been determined serologically to have blood type A according to the cat’s owner and one Donskoy with the A/b genotype indicative of blood type A had been determined serologically to have blood type AB. Retesting or further clinical investigation were not performed for these cats due to lack of availability.

After determining the suitability of 2019 typing panel, we based genetic blood typing on the variants: c.268T>A, (b^1^); c.179G>T, (b^2^); c.364C>T, (c—resulting in blood type AB); and c.1322delT (b^3^). The variants b^1^, b^2^ and c were observed widely distributed across breeds with frequencies of 12.6%, 1.6% and 1.5% in all cats, respectively (S4 Table). No more than two variants in total were found in any individual cat. Though generally uncommon in all cats, variant b^2^ was a major variant associated with blood type in the breeds Chartreux, Donskoy, Egyptian Mau, Minuet Longhair, Pixiebob, Siberian, Tennessee Rex, Tonkinese, Toybob, Turkish Angora and Turkish Van. Variant b^3^ was exclusively found in Ragdolls (16.9% allele frequency) and in a Ragdoll mix across the subset of 2,186 genotyped cats representing 69 breeds and varieties (S4 Table). As the data suggested that variant b^3^ is private to the Ragdoll population, blood type frequencies for each breed were estimated considering variants b^1^, b^2^ and c genotype (available across the entire study sample) for non-Ragdoll breeds and including b^3^ genotype results for the Ragdoll breed only.

Based on genetic blood type determination, blood type B was most common in the following five breeds or breed groups with >15 individuals tested: American Curl (40.4%), British Shorthair breed types (20.3%), Cornish Rex (33%), Devon Rex (30.3%) and Havana Brown (20%). In addition, the breeds in which the rare blood type AB was present with a frequency of >1% were European Shorthair (2.2%), Lykoi (1%), Scottish Fold (3.3%), RagaMuffin (3.4%), Ragdoll (2.2%) and Russian Blue breed types (1.5%). The proportions of type A, type B and type AB blood for each breed or breed group with >15 individuals tested are shown in Table 3 (breed groups listed in S6 Table).

**Table 3:** Genetically determined proportions of type A, type B and type AB blood for each breed or breed type with >15 individuals tested.

Breeds and breed types	No. of tested cats	Type A (A/A, A/b, A/c)	Type AB (c/b, c/c)	Type B (b/b)
Abyssinian breed types	214	100.0%	0.0%	0.0%
American Bobtail	44	84.1%	0.0%	**15.9%**
American Curl	47	59.6%	0.0%	**40.4%**
American Shorthair	49	98.0%	0.0%	2.0%
Bengal	1706	99.9%	0.1%	0.0%
Birman	174	89.1%	0.0%	**10.9%**
British Shorthair types	395	79.7%	0.0%	**20.3%**
Burmese breed types	161	98.8%	0.0%	1.2%
Chartreux	84	91.7%	0.0%	8.3%
Cornish Rex	106	66.0%	0.9%	**33.0%**
Devon Rex	446	69.7%	0.0%	**30.3%**
Donskoy	17	100.0%	0.0%	0.0%
Egyptian Mau	55	100.0%	0.0%	0.0%
European Shorthair	91	94.5%	2.2%	3.3%
Exotic Shorthair	68	95.6%	0.0%	4.4%
Havana Brown	30	80.0%	0.0%	**20.0%**
Highlander	231	99.6%	0.0%	0.4%
Khaomanee	21	100.0%	0.0%	0.0%
Korat	51	100.0%	0.0%	0.0%
LaPerm	35	100.0%	0.0%	0.0%
Lykoi	104	93.3%	1.0%	5.8%
Maine Coon	2121	99.2%	0.0%	0.8%
Manx breed types	46	97.8%	0.0%	2.2%
Munchkin breed types	109	97.2%	0.0%	2.8%
Norwegian Forest Cat	121	100.0%	0.0%	0.0%
Ocicat	76	100.0%	0.0%	0.0%
Oriental breed types	230	100.0%	0.0%	0.0%
Persian breed types	136	98.5%	0.0%	1.5%
Peterbald	17	100.0%	0.0%	0.0%
Pixiebob	33	93.9%	0.0%	6.1%
RagaMuffin	118	95.8%	3.4%	0.8%
Ragdoll	1115	96.1%	2.2%	1.7%
Russian Blue breed types	65	98.5%	1.5%	0.0%
Savannah	80	100.0%	0.0%	0.0%
Scottish Fold	90	87.8%	3.3%	8.9%
Scottish Straight	75	88.0%	0.0%	**12.0%**
Selkirk Rex	121	95.0%	0.0%	5.0%
Siamese breed types	233	99.6%	0.0%	0.4%
Siberian breed types	582	98.1%	0.0%	1.9%
Singapura	39	100.0%	0.0%	0.0%
Sphynx	547	92.1%	0.0%	7.9%
Tennessee Rex	35	100.0%	0.0%	0.0%
Tonkinese	41	100.0%	0.0%	0.0%
Toybob	56	87.5%	0.0%	**12.5%**
Turkish Angora	110	88.2%	0.0%	**11.8%**
Turkish Van	40	87.5%	0.0%	**12.5%**

### Factor XII Deficiency and Pyruvate Kinase Deficiency are widespread blood disorders in the cat population

Factor XII Deficiency is a widely distributed heritable trait in the domestic cat population [38]. Factor XII Deficiency is a clinical hemostatic defect that manifests as a prolonged activated partial thromboplastin time (aPTT) which would be observed in a presurgical coagulation assay, but does not require transfusions [39]. Two variants of the *F12* gene: c.1321delC and c.1631G>C, have been identified in a colony of inbred cats from the United States and in a litter of cats from Japan, respectively [40,41]. These variants are both considered common in the domestic cat [39]. The most severe aPTT prolongation is observed in cats homozygous for both variants [39]. In accordance with previous observations [39], we noted that the c.1321delC variant always co-segregates with c.1631G>C, while one or two copies of the latter can be inherited in the absence of the c.1321delC variant. The observed variant frequencies for the c.1321delC and c.1631G>C variants were 1.3% and 7% in all cats (Table 1). The frequency of the c.1631G>C variant was based on a subset of 2,186 genotyped cats, as the variant represented a more recent discovery added to the genotyping platform. The presence of the tested *F12* variants was found in the following breeds in which cases of clinical Factor XII Deficiency have been documented: Himalayan, Maine Coon, Manx, Munchkin, Oriental Shorthair, Persian, Ragdoll, Siberian, and Siamese. This supports the tested variants’ causal role in Factor XII Deficiency. Moreover, we discovered the presence of the c.1631G>C variant in the Turkish Angora, which is related to the Turkish Van breed, in which clinical cases of Factor XII Deficiency have been documented [39], but no representatives of this breed were in the subset of 2,186 cats screened for the presence of the c.1631G>C variant. Additionally, the tested *F12* gene variants were absent in Norwegian Forest Cats, in which the recently identified candidate variant c.1549C>T of *F12* gene could potentially be a more common cause of observed cases of Factor XII Deficiency [39]. In all, we identified the two variants of the *F12* gene present in 13 more breeds and breed types, and the c.1631G>C variant present alone in 19 additional breeds and breed types. The Tennessee Rex represents a breed with high frequency (>40%) for both variants observed together (Table 2). Finally, we obtained laboratory results from a 10-month-old intact female Maine Coon homozygous for the c.1321delC and c.1631G>C variants of *F12* gene. This individual showed a prolonged aPTT of >180 seconds (laboratory reference <13.4 seconds), confirming Factor XII Deficiency in a Maine Coon and further evidence of association between the tested variants and clinical signs.

Pyruvate Kinase Deficiency (PK-def) is an inherited anemia characterized by low levels of the pyruvate kinase enzyme. The insufficient presence of the pyruvate kinase enzyme causes red blood cells to break easily, resulting in hemolytic anemia. PK-def shows a marked clinical variability, including variation in the age of disease onset and severity [42,43]. A single nucleotide substitution (c.693+304G>A) in intron 5 of the *PKLR* gene, with a hypothesized impact on splicing, has been associated with the manifestation of PK-def in the Abyssinian and Somali breeds [43], but has also been previously reported in at least 15 additional cat breeds. Across the entire dataset the PK-def associated variant was found at 3.1% and 2.2% in the Abyssinian and Somali breeds respectively, and was also present in 21 additional breeds (Tables 2 and S4). Several cats from the Bengal, Maine Coon, and Maine Coon Polydactyl breeds were identified genetically at risk with two copies of the PK-def associated variant. Our veterinarians interviewed owners of ten Maine Coons (including two Maine Coon Polydactyls) and three Bengal cats. All the Maine Coons were 3 years old or younger at the time of interview. In three out of ten cases owners reported occurrence of at least one mild potential episode with clinical signs such as lethargy, anorexia, weight loss, and/or jaundice (S7 Table). One male Maine Coon, at the age of 1 year and 5 months, become severely ill with anorexia, lethargy, and significant weight loss (down to approximately 8 lbs from 17 lbs), another male had had episodes of mild lethargy and hyporexia, and the one female cat may have potentially manifested mild symptoms directly after giving birth. The three Bengal cats were between 2-years-9-months-old and 6-years-7-months-old at the time of interview. The owner of one female cat reported that the cat had stopped eating and seemed sick after an attempt to rehome, but fully recovered after returning to the breeder (S7 Table). We also interviewed the owner of a genetically affected Abyssinian cat. The 2-year-5-month-old male cat had four major episodes of illness each time starting with hyporexia and lethargy followed by anorexia, anemia, fever and jaundice, and each time had received significant veterinary care (S7 Table). Compared to the Abyssinian, all owner-reported episodes in the Maine Coon and Maine Coon Polydactyl are milder and should be considered preliminary results as they lack a complete blood count taken during the episode and PK-def diagnoses by a veterinarian.

### Autosomal dominant disease-associated variants are observed in additional breeds

We screened for four feline disease-associated variants that most closely follow an autosomal dominant mode of inheritance in clinical settings: Polycystic Kidney Disease (PKD) [14], two Hypertrophic Cardiomyopathy (HCM) variants [44,45], and Osteochondrodysplasia and Earfold [46].

Polycystic Kidney Disease (PKD) is a severe autosomal dominant (homozygous lethal) condition in which clusters of cysts present at birth develop in the kidney and other organs, causing chronic kidney disease which can lead to kidney failure [47,48]. PKD is caused by a stop codon in exon 29 of *PKD1*;c.9882C>A (published as c.10063C>A, coordinates updated to reference genome FelCat9), resulting in a truncated form of the gene, which was discovered in Persian cats with ~40% frequency in the Persian cat population worldwide [16–19]. Genetic testing was introduced into the breeding programs of Persians and some Persian-related cats. Our findings indicate that the overall frequency of the *PKD1* variant has decreased notably from what was previously reported in these breeds (Tables 2 and S4). However, the *PKD1* variant was identified in the Maine Coon, a breed in which it had not been previously documented in the peer-reviewed literature. Clinical manifestation of PKD in a genetically affected female Maine Coon, diagnosed at the age of 3 months, was confirmed after interviewing the cat’s owner and assessing associated diagnostic documentation including an ultrasound of the kidneys in which numerous, round, well-defined cysts were observed bilaterally throughout the renal cortex and medulla (S7 Table). This finding provides evidence that genetic screening for the *PKD1* variant in the Maine Coon is clinically relevant.

Hypertrophic cardiomyopathy (HCM) is the most common heart disease in domestic cats. Two independent variants of the *MYBPC3* gene c.91G>C p.(A31P) and c.2453C>T p.(R818W) (published as 2060C>T p.(R820W); coordinates updated to reference genome FelCat9) have been associated with HCM in the Maine Coon and Ragdoll breeds, respectively [44,45,49]. In the heterozygous state, the likelihood of developing clinical HCM early in life is very low. However, supporting the autosomal dominant mode of inheritance, regional diastolic and systolic dysfunction has been observed in heterozygous asymptomatic cats [50,51]. In the homozygous state, the development of HCM is highly likely in the Maine Coon with risk increasing with age [52]. Similarly, in the Ragdoll, heterozygous cats have a normal life expectancy, while homozygous cats are likely to have a shortened life span [53]. In the present study, we found the A31P and the R818W variants present in the heterozygous state in additional breeds (Table 2). Our veterinarians interviewed owners of four American Bobtail cats heterozygous for the R818Wvariant. One male had died suddenly at 5 to 6 years of age. Death was preceded by labored breathing over a few days, which the owner suspects was caused by a cardiac condition. However, no echocardiography had been applied to confirm a diagnosis of HCM (S7 Table). All three female American Bobtails at ages 4-years-and-4-months, 6-years-and-5-months and 10-years-and-5-months were still alive.

Osteochondrodysplasia and Earfold is a highly penetrant autosomal dominant condition caused by a missense variant (c.1024G>T) in the *TRPV4* gene resulting in congenital degenerative osteochondrodysplasia or “Scottish Fold Syndrome”, manifesting as skeletal deformities such as a short, thick, inflexible tail and malformation of the distal fore- and hindlimbs, which can lead to a stilted gait [46]. We observed one copy of the *TRPV4* variant in all 85 phenotype-confirmed Scottish Fold cats, and the *TRPV4* variant was absent in all 75 Scottish Straight cats tested. We also discovered one copy of the *TRPV4* variant in a crossbred cat resulting from the mating of a Scottish Fold and a Highlander. As the ear phenotype of the kitten was curled-back as seen in the Highlander breed, rather than folded forward as seen in the Scottish Fold, observation of the *TRPV4* variant was not entirely expected. However, the kitten did present a stiff and inflexible shortened tail, characteristic of *TRPV4* variant carriers. It therefore would appear that the yet unknown variant that causes the Highlander ear type masks the Scottish Fold ear phenotype caused by the *TRPV4* variant when the two variants are inherited together. In another recent study, a cat registered as an American Curl with curled ears was diagnosed with osteochondrodysplasia and genotypically showed one copy of *TRPV4* variant [54]. Thus, we report a second case of Osteochondrodysplasia in which the cat’s ear phenotype belied the presence of the causal variant.

### Molecular heterogeneity of feline hereditary retinal dystrophies

The disease-associated variants for retinal dystrophies screened in this study include *CEP290*, *KIF3B* and *AIPL1*. The *CEP290* variant is associated with late-onset Progressive Retinal Atrophy (PRA; discovered in the Abyssinian) and is present in many pedigreed breeds [55,56]. Here we document a frequency of 1.1% in all cats and have identified the presence of the *CEP290* variant in 20 additional breeds. The highest *CEP290* variant frequencies were observed in the Peterbald (26.5%) and in one of the additionally identified breeds, the Oriental Longhair (19.6%). The variant of the *KIF3B* gene, recently associated with an early-onset PRA; discovered in the Bengal) [57], was present in 6.9% of the Bengal breed (resulting in a frequency of 1.1% in all cats) (Tables 1 and 2 and S4). This variant was additionally discovered in the Highlander breed types and the Savannah. The *AIPL1* variant associated with PRA (discovered in the Persian) was the rarest variant associated with retinal dystrophies [58]; this variant was screened in a subset of 2,186 samples (including 5 Persian cats) and not observed at all (Tables 1 and S4). To pursue clinical validation of our findings, we recruited a 10-year-3-month-old female Oriental Longhair, homozygous for *CEP290*. Clinical validation was initiated with an owner interview, in which the owner reported no apparent changes in the cat’s behavior that were suggestive of vision loss (S7 Table). However, during an ophthalmic examination, a marked discoloration of pigmentation of the tapetal fundus with a slight vascular attenuation was noted, confirming the presence of retinal degeneration. This evidence suggests that rdAc-PRA may manifest clinically in the Oriental Longhair, and suggests vision may be retained longer than the previously reported 3–7 years [2], at least for this particular breed example.

### Feline MDR1 Medication Sensitivity associated with adverse medication reactions in the Maine Coon

Feline MDR1 Medication Sensitivity is a disorder associated with severe adverse reactions after exposure to medications that use the p-glycoprotein drug transporter. This genetic condition is caused by a two base pair deletion within exon 15 of the *ABCB1* gene resulting in abnormal p-glycoprotein [59]. While functional p-glycoprotein plays a significant part in the blood-brain barrier that prevents various drugs and chemicals in the bloodstream from entering the brain, a defective p-glycoprotein allows more drugs to cross this barrier, thus increasing the neurological effects of some medications. Severe macrocyclic lactone-induced neurologic toxicosis has previously been reported in cats homozygous for the MDR1 variant receiving either a subcutaneously administered dose of ivermectin or a topically administered eprinomectin-containing antiparasitic product labeled for cats [59,60]. We report the frequency of the *ABCB1* variant as 0.6% of all genotyped cats, in addition to the discovery of the variant in the Balinese, Maine Coon, Maine Coon Polydactyl, Ragdoll, Siamese and Turkish Angora breeds (Tables 1 and 2 and S4). Our veterinarians interviewed three owners of cats identified as homozygous for the MDR1 variant to assess their cat’s medical history (S7 Table). The cats consisted of a 1-year-4-month-old intact female Maine Coon, a 2-year-3-month-old intact female Ragdoll, and a 3-year-2-month-old intact male Maine Coon. All three cats had been administered topical flea medications (of varying brands) with no discernable side effects, however, none of the medications applied contained eprinomectin. One of the cats had undergone anesthesia and fully recovered, but reportedly was a bit more lethargic than expected.

### Genetic diagnosis plays a crucial role in the diagnosis of uncommon inherited disease

**Fig 1:**
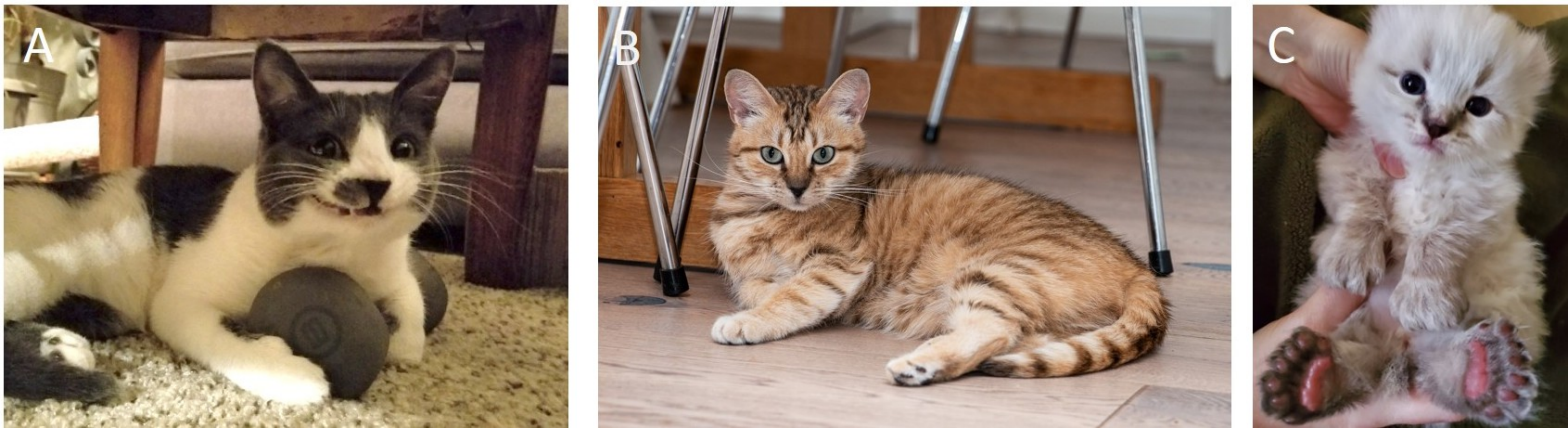
A) The signature “smile” of a cat with Myotonia Congenita; B) The rare coat color phenotype Amber in a random-bred cat from Finland; C) Polydactyly variant Hw also associated with extra toes in all four feet. Photo credit (from A to C): Kimberly Sullivan, Ari Kankainen, Samantha Bradley.

We identified a cat genetically affected with Myotonia Congenita, an uncommon recessively inherited disorder manifesting as an inability of the muscles to relax after contraction, which is caused by a variant in the *CLCN1* gene [37]. This is a sporadic condition that was discovered in a rescue domestic cat population in Winnipeg, Canada. While the variant was not identified in any pedigreed cats (which make up a large proportion of the study sample), we discovered two copies of the variant in a single non-pedigreed domestic cat in Oregon, United States. In the owner interview, we learned that the genetic diagnosis was crucial in assisting with clinical diagnosis (S7 Table). While this is an incurable condition, having the correct diagnosis helps ensure that the cat is getting appropriate supportive care. The owner confirmed that this cat, initially misdiagnosed with flea bite anemia at the age of 13 weeks, has a disease manifestation that includes fainting spells when startled, prolonged prolapse of the nictitating membrane, hypertrophic musculature, flattened ears, motor dysfunction, shortened gait, and limited range of motion in the jaw. The cat also shows a characteristic “smile” after a yawn or a meow due to delayed relaxation of the muscle in the upper lip as well as commonly has the paws protracted (Fig 1A). However, the owner mentioned that this cat, currently 4 years and 2 months old, is not drooling or showing any dental defects, which differs from the original disease description [37].

### Panel screening enables dissociation of variants with clinical disease

In this study we determined the c.140C>T p.(S47F) variant of the *UROS* gene to be present in 0.7% of cats across the entire study sample (Tables 1 and S4). This variant was previously discovered along with c.331G>A p.(G111S) in a cat manifesting Congenital Erythropoietic Porphyria (CEP) [61]. The manifestation of CEP includes distinctively stained brownish-yellow teeth that turn fluorescent pink under UV light. However, we did not observe the c.331G>A variant in any of the tested cats. While the original research revealed no cats carrying solely the c.140C>T variant, functional studies have shown that c.140C>T alone does not significantly alter the protein function [61]. Due to the high frequency of c.140C>T in some cat breeds and because some DNA testing laboratories offer tests for the two variants separately, our veterinarians reached out to the owners of cats homozygous for only the c.140C>T variant resulting in five cases: a 1-year-4-month-old intact female RagaMuffin, a 1-year-5-month-old intact male RagaMuffin, a 1-year-7-month-old intact female Siberian, a 2-year-10-month-old intact female Singapura and a 3-year-7-month-old intact male Toybob. None of these cats had clinical signs suggestive of CEP. Thus, the existing evidence strongly suggests that c.140C>T is a benign variant when it is not inherited along with c.331G>A.

Mucopolysaccharidosis Type VI (MPS VI), a lysosomal storage disease caused by a deficiency of N-acetylgalactosamine-4-sulfatase (4S), is another disease in which the roles of two variants, independently inherited in this case, have been under discussion [62]. While the MPS VI variant c.1427T>C p.(L476P) of the *ARSB* gene is associated with severe disease [63], the c.1558G>A p.(D520N) variant of the *ARSB* gene is sometimes referred to as the “mild” type [64]. However, it is necessary for the D520N variant to be inherited as a compound heterozygote with one copy of the L476P variant for the disease to manifest in its mild form. We have elected to call the D520N variant MPS VI Modifier. The D520N variant is present in large numbers of cats, with a frequency of 2.5% (Tables 1 and S4). It is also the major allele in the Havana Brown, with an allele frequency of 76.7%. Additionally, we confirm that L476P is a scarce variant, and this study cohort did not identify any cats with MPS VI variant in concordance with previously described observations [62]. The high variant frequency of the benign MPS VI Modifier and very low frequency of the MPS VI variant (absent in many breeds) further confirm that breeding to avoid MPS VI should concentrate solely on managing the L476P variant.

### Appearance-associated variants distributed across breeds and breed types

In this study sample of mostly pedigreed cats (94.4%), the ancestral form of the appearance-associated (trait) variant was the major allele, except for the *ASIP* gene in which the derived allele a (non-agouti/solid color) [65] showed a 56.2% variant frequency in all cats (S4 Table; include all within breed variant frequencies). The rarest derived allele observed was the e allele of the *MC1R* gene associated with the coat color Amber (discovered in the Norwegian Forest Cat) [66], which was observed in the Norwegian Forest Cat and one random-bred cat from Finland. The random-bred cat was homozygous for the derived allele with pictures confirming the expression of the Amber coat color (Fig 1B).

Expectedly, a high number of breeds and breed types shared the same trait-associated variants in common (S4 Table). For example, the c.904G>A variant of the *TYR* gene resulting in Colorpoints (discovered in the Siamese) [67], is also the cause of the Colorpoint phenotype in the Birman, Himalayan (a colorpoint type of Persian cats) Ragdoll, Neva Masquerade (a colorpoint type of Siberian), Ragdoll and Toybob. The derived variant is also present in >30% of the representatives of American Curl, Bengal, British Longhair, Cornish Rex, Donskoy, Highlander, LaPerm, Minuet, Munchkin, Oriental Longhair, Oriental Shorthair, Peterbald, Sphynx and Tonkinese. Genotyping results of trait-associated variants showed overall concordance with observed phenotypes, strongly supporting the variants’ causality. For the two variants White Spotting and Dominant White, which are a result of a full or partial feline endogenous retrovirus (FERV1) insertion into *KIT* gene [68], we had a genotyping assay that detected if either one of the variants was present requiring combination of genotype with the cat’s phenotype for result interpretation. The White Spotting variant, which we believe to be more ancestral of the two, was much more common in cats than the Dominant White phenotype. In the Turkish Van breed (the name for the breed comes from the “Van pattern” where the colored areas are restricted to the area around the ears with a few additional spots), all the cats had two copies of the White Spotting based on the phenotypic observations. In other breeds with phenotypes, both one and two copies of the White Spotting manifested as a variable coverage of white areas on the cat and based on the cat’s appearance, it was not possible to determine how many copies of the White Spotting was present. However, we observed that in cats with white paws (even when the rest of the legs had color), at least one copy of the White Spotting was present in all breed backgrounds. The only exception were the Birman cats, in which the breed-defining white paws (aka Gloves) are associated with the two adjacent missense changes of the *KIT* gene [69]. This derived variant had a frequency of 95.6% in the Birman breed and the cats manifested white paws in the absence of White Spotting. Interestingly, we also observed the derived variant as a minor allele in many breeds, in which white paws are not seen. We identified two copies of the Gloves variant present in individuals of the Chartreux, Highlander, Maine Coon, Ragdoll and Siberian breeds, in which they were not associated with Gloving based on photographic evidence and owner reports. Of the phenotyped individuals some white areas of hair were seen on the belly or toes in (3/17) Maine Coons and (1/2) Siberian cats. Our findings are supportive of a potential additional candidate locus that may play a role in the regulation of the Gloves phenotype [70].

Moreover, we observed the Hairlessness variant (discovered in the Sphynx) [71], the Shortened Kinked Tail variant (discovered in the Japanese Bobtail) [72] and the three tested Short tail variants (discovered in the Manx cat) [73] result in their expected phenotypes, but also not explain all observed bald and short tail phenotypes. The c.816+1G>A variant in the *KRT71* gene which is known to result in the hairless phenotype of the Sphynx cat had a variant frequency of 74.7% in the Sphynx breed [71] (S4 Table). Compound heterozygotes for the Sphynx c.816+1G>A variant with the Devon Rex associated curly coat variant are also hairless [71]. We also identified 22 hairless Sphynx cats without any copies of the Hairlessness variant, suggesting that an additional unknown variant in the same or another gene entirely causes the hairless phenotype in some cats of this breed. We also confirmed that the Hairlessness variant was not present in the Donskoy and Peterbald breeds, which also represent hairless phenotypes. It was recently suggested that a novel 4 base pair variant of the *LPAR6* gene identified in a Peterbald cat potentially results in a hairless coat phenotype as a homozygote or as a compound heterozygote with the c.250_253_delTTTG variant in the *LPAR6* gene which causes the curly coat of the Cornish Rex [74]. We show a variant frequency of 25% for the Cornish Rex coat variant in the Donskoy, but did not identify any Cornish Rex coat variant carriers among the 17 tested Peterbald cats of this study.

The c.5T>C variant in the gene *HES7* associated with a shortened and kinked tail in the Japanese Bobtail [72] was observed as homozygous in all Japanese Bobtail cats of this study, with the derived variant also prevalent in the Kurilian Bobtail and Mekong Bobtail. In additional bobtail breeds, Cymric and Toybob, a few individuals were also observed with the derived variant (S4 Table). All studied cats with one or more copies of *HES7* variant and available phenotypic information presented with a shortened tail.

We genotyped three out of four known variants (c.995delT, c.1166delC, c.1196delC) (published as 998delT, c.1169delC and c.1199delC; coordinates updated to reference genome FelCat9) of the *T* gene (discovered in the Manx) causing shortened tail [73]; the fourth T variant (c.995_1011dup;1011_1014del on FelCat9 reference genome, published as 998_1014dup17delGCC), which has only been previously observed in one cat, could not be genotyped. We found one of the three tested variants in all the Pixiebobs (including longhair variant), 9/30 (30.0%) Manx and 7/14 (40.0%) Cymric (a Manx type with longhair). The derived variants of the T-box were always observed in the heterozygous state, further suggesting that in the homozygous state these variants are lethal in utero [73], and therefore in the mating of two short-tailed cats 25% of cats conceived are born with longtail. Long-tailed Manx and Cymric cats are part of the breeding programs and because there was no phenotype information available for all tested Manx and Cymric cats, it could not be estimated if the tested three variants explained all short tail phenotypes in these two breeds. In addition, in line with previous study [73], the variant c.995delT of the *T* gene was also observed in American Bobtails (combined group of both Longhair and Shorthair variants). The tested Manx shorttail variants were also remarkably common in the Highlander breed, in which there is believed to be a different cause for the short tail phenotype. In this study cohort, 40.6% (94/231) of the Highlanders had one of the tested short tail variants (discovered in the Manx) (S4 Table). The photographic evidence in the American Bobtail and Highlander breeds confirm several short-tailed cats that were not carrying any known bobtail variant (S4 Table).

Lastly, we found that of the three tested polydactyly (extra toes) associated variants, the Hw variant of the *LMBR1* gene was predominantly observed in polydactylous cats, including the Maine Coon, Pixiebob and Highlander breed types, and some non-pedigreed cats from North America. Both heterozygous and homozygous cats observed, and based on the photographic evidence presenting with four (normal number of digits) to seven toes per paw. Higher variant penetrance was seen in homozygous cats, which is in line with previous observations [75]. However, extra toes did not manifest solely in the front feet as previously reported; photographic evidence and owner-provided details revealed the presence of extra toes on all four feet (Fig 1C), which was formerly considered to be characteristic of the UK1 and UK2 variants only [75]. We also confirmed that a non-pedigreed cat with two copies of the UK1 variant had two extra digits on each paw, per the owner’s description. Various owner-reported cases of polydactyl cats testing negative for the screened variants were also noted. Such cats are likely to be carrying additional variants of the *LMBR1* gene or other not yet identified locus.

### Genome-wide analysis of genetic diversity demonstrates differences between and within cat breeds

**Fig 2:**
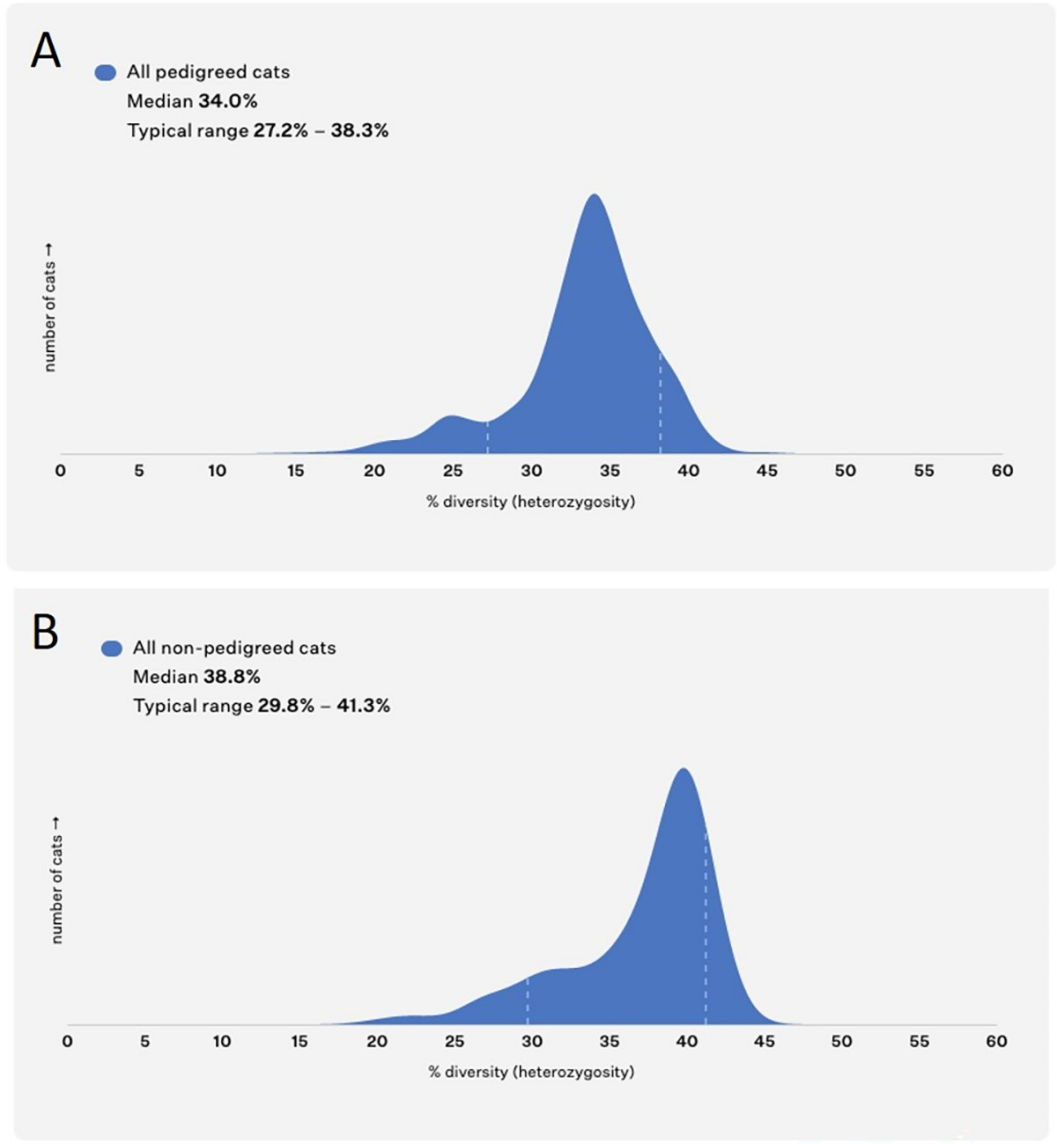
The median genetic diversity in pedigree and non-pedigreed cat populations with typical range (the 10th and 90th percentile).

The entire data set of 11,036 samples was genotyped for 7,815 informative SNP markers distributed across the genome. In the pedigreed cat population, the median heterozygosity was 34.0% and the typical range (defined as the 10th and 90th percentile) was 27.2%-38.3%, in the non-pedigreed population, the median heterozygosity was 38.8%; and the typical range was 29.8%-41.3% (Fig 2). The median heterozygosity was calculated for 60 breeds and breed types that were represented by at least 15 individuals in the dataset (S8 Table). The most diverse breeds include three of the newer cat breeds: the short-legged Munchkin, produced from a sibling mating followed by regular non-pedigreed cat outcrosses [7,67]; the Highlander, a crossbreed of two recent experimental hybrid cat breeds the Desert Lynx and Jungle Curl; and the Lykoi breed founded by unrelated cats expressing hypotrichosis, whose unique sparse and roaned coat phenotype may be caused by any of six different variants of the *HR* gene from six independent lineages found in four different states of the United States, Canada and France [76]. The heterozygosity levels of the European Shorthair, Norwegian Forest Cat, Siberian, and Manx, which were developed from the local domestic populations that likely had a larger diversity in the founder population, were above average compared with the entire pedigreed cat population. The lowest median heterozygosity measures in any pedigreed cat population were observed in the Burmese, Birman, Havana Brown, Korat, Singapura and breeds of the Siamese group (such as Balinese, Siamese and Oriental Shorthair), in line with previous observations [30]. A full breakdown of the diversity levels per breed can be found in S8 Table.

## Discussion

In the largest DNA-based feline study cohort to date, a custom genetic panel screening test was used to determine blood type, disease and phenotypic trait heritage, as well as the relative genome-wide genetic diversity in 11,036 domestic cats.

One specific area of focus was blood type determination, which is important in cat breeding due to its link with neonatal isoerythrolysis, a significant cause of fading kitten syndrome and neonatal death if the blood types of breeding pairs are not appropriately matched. Here we show that cats with blood type B are present in a vast majority of the breeds. We identified 11 breed types in which at least one out of ten cats in the population were blood type B. We also observed the rare blood type AB, previously described with notable prevalence only in the Ragdoll, to be present in the European Shorthair, Scottish Fold breed types, and the RagaMuffin. Based on the proposed DNA genotyping scheme for purpose-bred cats, and serological blood type based on the 220 pedigreed cat results available for analysis in this study, we confirm a high concordance between blood type determination. However, while there are several studies confirming genotypes associated with blood type AB [20,23], the genetic blood type determination and immunological blood type determination for two cats were unconcordant. In a recent study [77], type AB cats which carried one copy of a variant resulting in blood type B, had a higher level of Neu5Ac present that could depending on the technologies used impact results. This provides further justification for the supportive role of the DNA-based blood type determination approaches in breeding and veterinary care, provided that the appropriate carefully validated genetic variants are assayed. However, additional genetic variants of the *CMAH* gene have been identified that may play a role in blood type determination in domestic shorthair and stray cats [23,77], and further investigations are warranted.

Panel screening of disease-associated variants provides potential clinically relevant information in addition to blood type determination. We show that the genomic data available through routine genetic screening of any cat today can assist in disease diagnosis, treatment, and preventative care. Through the comprehensive investigation of variant allele frequencies in this study cohort, we re-evaluate and provide updated variant frequency information compared to estimates provided in conjunction with the original variant discoveries. We identify several disease-associated variants as common and widely spread across breeds, suggesting that they are ancient in their origin. The most common disease-associated genetic variants of this study are Factor XII Deficiency, PK-def and rdAC-PRA. Factor XII deficiency is likely non-pathological but nevertheless a major clinical differential of genetically affected cats that despite the prolonged aPTT do not manifest extensive bleeding or require transfusion [39–41]. The c.707-53G>A variant of *PKLR* (published as c.693+304G>A; coordinates updated to reference genome FelCat9) associated with PK-def in the Abyssinian and Somali breeds is also found across multiple breeds in which genetic testing for PK-def has been employed [43], though the clinical manifestation of PK-def in these additional breeds has not been documented in the scientific literature. This may be partly explained by the episodic nature of this disease and its remarkable variability in expressivity, making it diagnostically challenging [42,43,78], and the fact that the advent of genetic testing made it rare. Clinical diagnosis using appropriate clinical examination of cats that are genetically predisposed to PK-def with symptoms is still needed to confirm the variant’s penetrance in different breed backgrounds. The variant rdAC-PRA (discovered in the Abyssinian) causes a late onset disease with documented variant penetrance across several breeds [55]. Today, the highest allele frequencies are seen in the cat breeds of the Siamese group, which also have decreased genetic diversities. The management of rdAC-PRA in these breeds requires a well-planned breeding program that includes keeping carriers in breeding and using them responsibly.

Extensive DNA panel screening also allows investigation of putative or candidate variants that have been previously described in a limited number of samples or in a single domestic cat. We report Feline MDR1 Medication Sensitivity to be present in 126/11,036 (1.1%) cats of this study in representatives of the breeds Balinese, Maine Coon, Maine Coon Polydactyl, Ragdoll, Siamese, Turkish Angora, and non-pedigreed cats. This variant was also recently discovered in 4% of domestic shorthairs, Ragdoll, Russian Blue and Siamese [59,60]. The existence of Feline MDR1 Medication Sensitivity is not yet well known in the veterinary community, but a recent study shows that affected cats develop severe neurologic symptoms even when treated with eprinomectin-containing products labeled for use in cats and administered according to label instructions [60]. When treating cats with this variant, precautions to those recommended for dogs with MDR1 are advised. We report three rare genetic variants associated with disorders present in the heterogeneous non-pedigreed cat study sample, of which a discovery of Myotonia Congenita confirmed clinical diagnosis for a cat that ensures its appropriate treatment and care.

Our data indicate that known disease-associated variant frequencies are now lower for many conditions (GM2 and Hypokalemia in Burmese, Glycogen Storage Disease in Norwegian Forest Cats, HCM and Spinal Muscular Atrophy in Maine Coon, HCM in Ragdoll and PKD in Persian) compared to the frequencies at the time of their discovery, perhaps reflecting change over time within the breed, presumably due to genetic testing combined with informed breeding selection. For example, the *PKD1* variant, which initially affected nearly 40% of Persian cats [14], was found at higher frequency in breeds with Persian background or non-related breeds than it was in the Persian breed. In fact, none of the Persian cat samples in this study had the *PKD1* variant. Additional recent studies indicate that the prevalence of PKD in Persian cats of Iranian origin continues to be high [71], while *PKD1* is common in pedigreed and non-pedigreed cats in Iran, Japan and Turkey [11–13]. Moreover, we rarely observed the known autosomal dominant disease-associated variants for HCM (discovered in the Maine Coon) and HCM (discovered in the Ragdoll), in the homozygous state which confers significantly higher risk for disease development compared to heterozygotes [52,53]. Similarly, in this study sample the Scottish Fold cats were all heterozygous for the autosomal dominant Osteochondrodysplasia and Earfold which may result in a milder phenotype [46]. Thus, this data reveal that there is an active DNA testing culture in the feline fancy used to carefully manage disease predisposing variants that are known in the breed. Our study cohort was biased towards breeding animals of the breeders with active DNA testing routines and interests, which may account for some disease-associated variants being seen less commonly than would be observed in randomly selected samples.

Utilizing the DNA panel testing approach has many justifications, especially in felines in which many breeds are the outcome of different cross-breedings and the molecular disease heritage introduced in the resulting breed is unlikely to be fully known. Here we show identification of 13 disease-associated variants in additional breeds and breed types that these variants have not been previously reported in, including breed types that have been underrepresented in many previous studies such as Balinese, Highlander, Munchkin, Minuet, Pixiebob, Savannah, Tennessee Rex and Turkish Angora. Here we also provide variant allele frequencies for breed types (varieties of the breed) separately, according to registry information reported by the owner. While our findings in additional breeds include several that can be explained by the history of known cross-breeding, we also report genetic diseases such as HCM (discovered in the Ragdoll) in American Bobtail and PK-def in Maine Coon Polydactyl and Pixiebob are observed more commonly in other breed backgrounds than in the original breeds in which they were discovered, likely due to lack of awareness and inadvertent selection. Comprehensive genetic testing has a very important role in a new breed’s development, in identifying which disease variants have been introduced in conjunction with breed formation. For example, in the Highlander, one of the most genetically diverse breeds, we found the presence of disease-associated variants for HCM (discovered in the Ragdoll), PK-def, rdAC-PRA, PRA (discovered in the Bengal) and Spinal Muscular Atrophy (discovered in the Maine Coon) which represent targets for genetic screening and management in the breed. In this study we mainly focused on genotyping pedigreed cats and had a relatively small sample size of non-pedigreed cats. We nevertheless discovered 13 disease-associated variants in non-pedigreed cats, highlighting the relevance of genetic screening for diagnostic purposes in this population as well.

This study cohort provides an extensive investigation of disease-associated variant heritage across pedigreed cat populations. Large scale screening studies of isolated subpopulations of a species such as pedigreed cats hold great value as a secondary independent tool for validating original discoveries as often they are made by focusing on a limited number of individuals from a single breed. Investigations that extend beyond the original discovery breed enable researchers to conclusively understand the causal relationships between variants and diseases. For each disease-associated variant discovered in additional breeds in this study, our follow up investigations applied a similar validation protocol as previously recommended for dogs by combining genotype information with clinical information collected and evaluated by veterinarians to assess variant manifestation in different breed backgrounds [32]. These clinical phenotype evaluation studies are crucial to ensure genetic counseling information that truly offers solutions to improve the health of cats. Here we offer further supporting evidence for the causal relationship between several disease-associated variants (*F12*, *PKD1*, *TRPV4*, *CEP290*, *ABCB1* and *CLCN1*) and their clinical manifestations. Our findings further highlight that the *PKD1* variant should be seen as a potential genetic cause of PKD in any breed, such as the previously documented Neva Masquerade [79], Chartreux [80], or the Maine Coon as reported in this study. Moreover, we found that Scottish Fold crosses are of particular concern, as heterozygous cats also manifest Osteochondrodysplasia and the presence of *TRPV4* derived allele cannot be reliably detected from the ear type if the cat has ear Curl, as also shown by others previously [54]. Moreover, the heterozygosity for *TRPV4* in chondrodysplastic (short-legged) cats can be a cause of severe pain. Additionally, here we present a preliminary indication of a phenotype for PK-def in Maine Coons (pending complete clinical examination and diagnosis) and a potential case for *MYBPC3* variant (R818W; discovered in the Ragdoll) contributing to a suspected cardiac cause of death in an American Bobtail, which call for further investigations. This study was fueled by the cat community and individual breeders’ willingness to provide phenotype information, clinical documentation, and participate in veterinary examinations. In the future, more systematic methods to collect phenotype information to associate with genotype information could be obtained through surveys or by implementing genetic testing as a part of the health care plan for the cat, allowing direct connection between genotype information and medical records. Finally, after evaluation, we report two associated disease variants that have little value as markers for genetic disease. Both the c.140C>T variant of the *UROS* gene previously co-segregating with a second variant associated with CEP and the c.1558G>A variant of *ARSB* gene were found to be the major variants in some breeds without any health impact. We advise DNA testing laboratories to discontinue offering a test for CEP based solely on the use of the c.140C>T variant, The MPS VI Modifier. Moreover, like previous investigations [62], we further emphasize that prevention of MPS VI should focus entirely on managing the c.1427T>C variant in the cat population. The MPS VI Modifier is an asymptomatic variant that contributes to a mild phenotypic expression of disease in compound heterozygotes [62–64], suggesting that selecting against the c.1558G>A variant is not justified or recommended, as it would also reduce the genetic variation in the breed.

The appearance of the cat is influenced by various genes which are often monitored by genetic testing to inform breeding pair selection. In this study, all cats were tested for 26 appearance-associated variants. Information was provided on the frequency at which the trait variants are encountered across breeds, to explain the observed phenotypes. The same trait variants influencing coat color/type and morphology are highly frequent in cats of various breed backgrounds, providing evidence of likely causality. However, we note that the *KIT* gene variant associated with breed-characteristic white feet in Birman cats [69], according to photographic evidence, is a low penetrance variant in various other breeds. Moreover, while most of the trait phenotypes were explained by the known variants, the previously discovered variants associated with shortened tail, extra digits, and hairlessness could only explain the presence of some of these phenotypes.

Our analysis of genetic diversity in cat breed populations shows a wide range of diversity levels within and between breeds. We found evidence that, as expected, more recently formed breeds with a more significant number of founding individuals and breeds allowing continued outcrossing tend to have the greatest diversity levels. Maintaining diversity in closed populations is challenging, and the use of outcrossing may help maintain and potentially increase diversity levels if widely adopted. The importance of preserving diversity for health and vigor has been widely documented [81–83].

In conclusion, we demonstrate that several feline disease-associated variants are more widespread across cat breeds and breed types than previously reported, with both dominant and recessive Mendelian disease-associated variants observed in additional breeds and often at higher allele frequency than the breeds in which they were originally discovered. This, in part, demonstrates the effectiveness of proactive genetic testing, which has reduced disease-associated variant frequencies in notably affected breeds over time. We have also shown that some disease-associated variants are very rare and limited to specific breeds. We report the prevalence at which the three clinically relevant feline blood types occur within breeds and breed types and provide trait variant frequencies across the feline population. We have combined genotype information with phenotypic information to investigate and re-evaluate causality in different breed backgrounds, confirming causal relationships for some variants and weak evidence of penetrance for other variants. In summary, genetic testing can be used to inform breeding decisions aiming to prevent genetic disease, while a concurrent goal should be to maintain genetic diversity in a breed’s population, helping to sustain the breed. As more cats are genotyped, we will learn more about feline variant heritage in the broader domestic cat population, leading to improved health care advice for all cat owners. Direct-to-consumer tests help to further raise awareness of various inherited conditions in cats, provide information that owners can share with their veterinarians, and in time, as more genotypic and phenotypic data are collected, will enable the genetics of common complex feline disease to be deciphered, paving the way for personalized precision healthcare with the potential to ultimately improve welfare for all cats.

## Supporting information

S1 Table The summary of 11,036 tested pedigreed and non-pedigreed cats. (XLSX)

S2 Table Tested disease and trait associated variants. (XLSX)

S3 Table All tested disease and trait genotype data for 11,036 tested cats. (XLSX)

S4 Table All tested disease and trait variant frequencies for 11,036 tested cats. (XLSX)

S5 Table Immunological and genetic determination of the blood type for 220 cats. (XLSX)

S6 Table Clustered breed information for representing the proportions of the different blood types with >15 tested individuals. (XLSX)

S7 Table Phenotypic Information for Evaluation of Nine Disease-associated Variant’s Penetrance in 31 cats. (XLSX)

S8 Table Genetic diversity for all breeds with >15 individuals tested. (XLSX)
